# Supplementary material for: The interactome of intact mitochondria by cross-linking mass spectrometry provides evidence for coexisting respiratory supercomplexes
Source: Mol Cell Proteomics. 2017 Dec 8;17(2):216–32. doi: 10.1074/mcp.RA117.000470 (PMC5795388; doi:10.1074/mcp.RA117.000470)
Supplement: Supplemental Data [file supp_RA117.000470_133922_0_supp_23978_fzffwf.zip › spectra_annotation/mito_spectra_annotation/41-1-2-1-2-2.pdf]

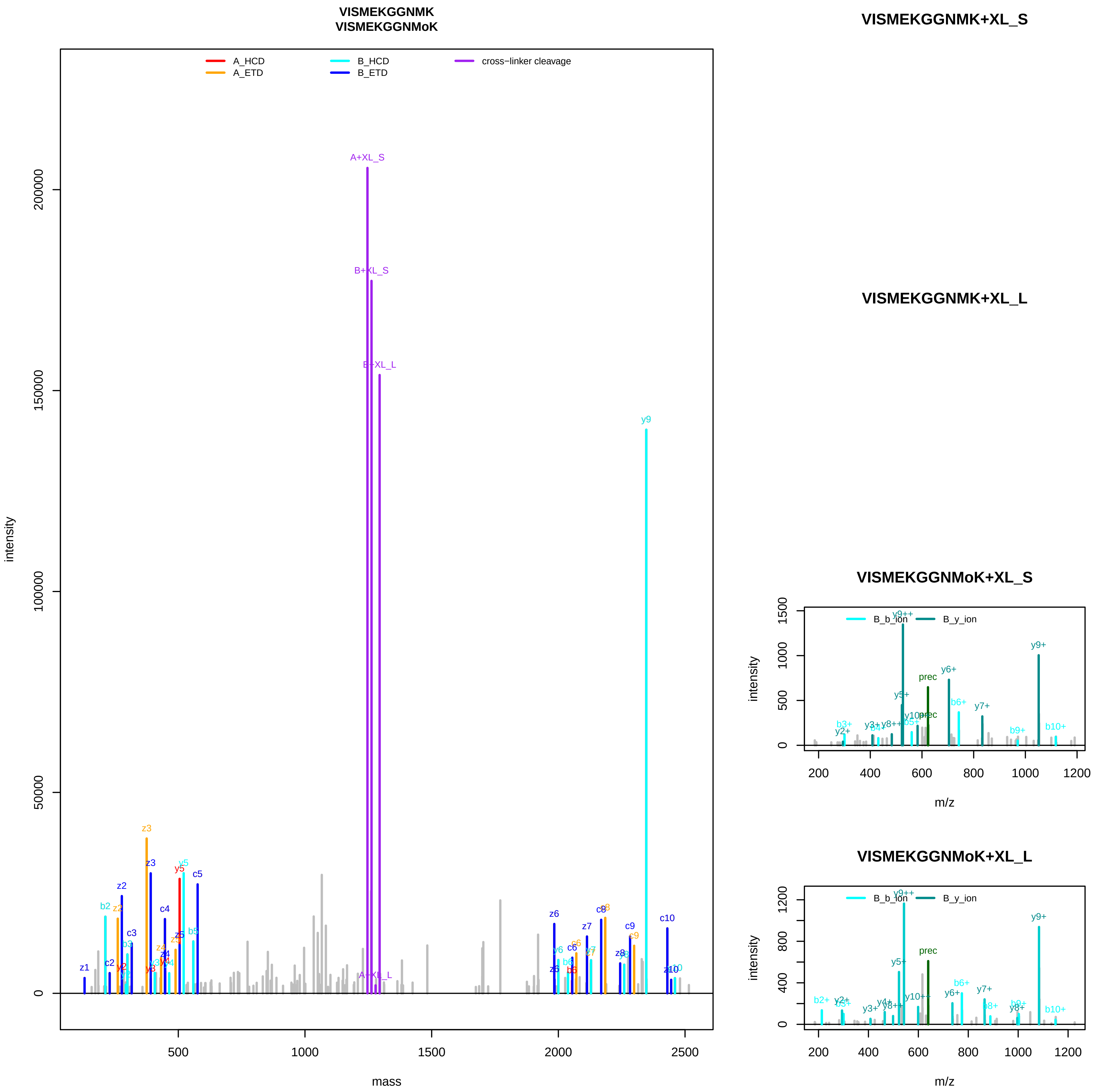
VISMEKGGNMK
VISMEKGGNMoK
A_HCD
A_ETD
B_HCD
B_ETD
cross−linker cleavage
A+XL_S
B+XL_S
B+XL_L
A+XL_L
y9
z1 c2 y2 z2 b2 z2 b3 c3 y3 z3 z3 c4 z4 y4 y3 z4 y4 z5 z5 y5 y5 c5 b5
z6 y6 z6 b6 c6 c6 b6 z7 y7 c7 c8 c8 z8 y8 c9 c9 c10 z10 b10
0
50000
100000
150000
200000
intensity
500
1000
1500
2000
2500
mass
VISMEKGGNMK+XL_S
VISMEKGGNMK+XL_L
VISMEKGGNMoK+XL_S
B_b_ion
B_y_ion
y9++ y9+ prec y6+ y5+ b6+ y10++ prec y7+ b3+ y2+ y3+ b4+ y8++ b5+ b9+ b10+
0
500
1000
1500
intensity
200
400
600
800
1000
1200
m/z
VISMEKGGNMoK+XL_L
B_b_ion
B_y_ion
y9++ y9+ prec y5+ b6+ y7+ y6+ b2+ y2+ b3+ y3+ y4+ y8++ y10++ b8+ b9+ y8+ b10+
0
400
800
1200
intensity
200
400
600
800
1000
1200
m/z
